# Cross-Polarization Optical Coherence Tomography for Clinical Evaluation of Dermal Lesion Degrees in Vulvar Lichen Sclerosus

**DOI:** 10.17691/stm2023.15.1.06

**Published:** 2023-01-28

**Authors:** A.L. Potapov, M.M. Loginova, A.A. Moiseev, S.G. Radenska-Lopovok, S.S. Kuznetsov, I.A. Kuznetsova, N.N. Mustafina, I.K. Safonov, N.D. Gladkova, M.A. Sirotkina

**Affiliations:** PhD Student, Laboratory Assistant, Scientific Laboratory of Optical Coherence Tomography, Institute of Experimental Oncology and Biomedical Technologies; Privolzhsky Research Medical University, 10/1 Minin and Pozharsky Square, Nizhny Novgorod, 603005, Russia;; Junior Researcher, Scientific Laboratory of Optical Coherence Tomography, Institute of Experimental Oncology and Biomedical Technologies; Privolzhsky Research Medical University, 10/1 Minin and Pozharsky Square, Nizhny Novgorod, 603005, Russia; PhD Student, Institute of Biology and Biomedicine; National Research Lobachevsky State University of Nizhni Novgorod, 23 Prospekt Gagarina, Nizhny Novgorod, 603950, Russia;; Senior Researcher, Laboratory of Highly Sensitive Optical Measurements; Federal Research Center Institute of Applied Physics of the Russian Academy of Sciences, 46 Ulyanova St., Nizhny Novgorod, 603950, Russia;; Professor, Institute of Clinical Morphology and Digital Pathology; I.M. Sechenov First Moscow State Medical University (Sechenov University), 8/2 Malaya Trubetskaya St., Moscow, 119991, Russia;; Professor, Head of Pathological Department; Nizhny Novgorod Regional Clinical Hospital named after N.A. Semashko, 190 Rodionova St., Nizhny Novgorod, 603126, Russia; Head of the 2^nd^ Gynecological Department; Nizhny Novgorod Regional Clinical Hospital named after N.A. Semashko, 190 Rodionova St., Nizhny Novgorod, 603126, Russia Associate Professor, Department of Obstetrics and Gynecology; Privolzhsky Research Medical University, 10/1 Minin and Pozharsky Square, Nizhny Novgorod, 603005, Russia;; Obstetrician-Gynecologist; Nizhny Novgorod Regional Clinical Hospital named after N.A. Semashko, 190 Rodionova St., Nizhny Novgorod, 603126, Russia; Obstetrician-Gynecologist; Nizhny Novgorod Regional Clinical Hospital named after N.A. Semashko, 190 Rodionova St., Nizhny Novgorod, 603126, Russia; Professor, Head of the Scientific Laboratory of Optical Coherence Tomography, Institute of Experimental Oncology and Biomedical Technologies; Privolzhsky Research Medical University, 10/1 Minin and Pozharsky Square, Nizhny Novgorod, 603005, Russia;; Director of the Institute of Experimental Oncology and Biomedical Technologies; Privolzhsky Research Medical University, 10/1 Minin and Pozharsky Square, Nizhny Novgorod, 603005, Russia;

**Keywords:** cross-polarization OCT, OCT attenuation coefficient, co-polarization, cross-polarization, vulva, vulvar lichen sclerosus

## Abstract

**The aim of the study** was to identify different degrees of dermal lesions in vulvar lichen sclerosus (VLS) using cross-polarization optical coherence tomography (CP OCT) based on attenuation coefficient to detect disease early manifestations and to monitor the effectiveness of treatment.

**Materials and Methods:**

The study included 10 patients without pathology and 39 patients with VLS diagnosed histologically. CP OCT was performed *in vivo* on the inner surface of the labia minora, in the main lesion area. From each scanning point, a 3.4×3.4×1.25-mm3 3D data array was obtained in 26 s. CP OCT examination results were compared with histological examination of specimens stained with Van Gieson’s picrofuchsin.

Quantitative analysis of OCT images was performed by measuring the attenuation coefficient in co-polarization and cross-polarization. For visual analysis, color-coded charts were developed based on OCT attenuation coefficients.

**Results:**

According to histological examination, all patients with VLS were divided into 4 groups as per dermal lesion degree: initial (8 patients); mild (7 patients); moderate (9 patients); severe (15 patients). Typical features of different degrees were interfibrillary edema up to 250 μm deep for initial degree, thickened collagen bundles without edema up to 350 μm deep for mild degree, dermis homogenization up to 700 μm deep for moderate degree, dermis homogenization and total edema up to 1200 μm deep for severe degree.

Pathological processes in dermis during VLS like interfibrillary edema and collagen bundles homogenization were visualized using CP OCT method based on values of attenuation coefficient in co- and cross-polarization channels. However, CP OCT method appeared to be less sensitive to changes of collagen bundles thickness not allowing to distinguish thickened collagen bundles from normal ones with enough statistical significance. The CP OCT method was able to differentiate all degrees of dermal lesions among themselves. OCT attenuation coefficients differed from normal condition with statistical significance for all degrees of lesions, except for mild.

**Conclusion:**

For the first time, quantitative parameters for each degrees of dermis lesion in VLS, including initial degree, were determined by CP OCT method allowing to detect the disease at an early stage and to monitor the applied clinical treatment effectiveness.

## Introduction

Vulvar lichen sclerosus (VLS) is an inflammatory disease of the vulvar and perianal skin characterized by a chronic and recurrent course. The disease etiology is poorly understood, but an autoimmune pathogenetic mechanism is assumed [1]. VLS most often affects postmenopausal women, causes exhausting clinical symptoms (itching, burning) which reduce quality of life [2]. Long-term course of the disease inevitably leads to destructive scarring of the vulvar skin; its anatomical structures are destroyed causing serious functional disorders of the urinary and reproductive systems [3]. Risk of developing squamous cell cancer exists, increasing with VLS long course and late diagnosis. Different researchers estimate cancer development absolute risk as 0.2 to 4.0% [4]. Early diagnosis of the disease followed by long-term therapy with topical corticosteroids can prevent scarring and malignization and help to control symptoms [5]. However, untimely diagnosis of VLS is still a common phenomenon.

Early diagnosis is difficult, as VLS clinical symptoms are mild and nonspecific. In this case, a biopsy is necessary to clarify the diagnosis [6]. Biopsy as an invasive examination method has a number of limitations, and gynecologists do not often use it due to clear clinical indications unavailability or to patient’s refusal.

In VLS, main pathological processes affect dermis’ extracellular matrix. Dermis changes, in turn, are pathognomonic and used as criteria for histologic diagnosis [7]. In our previous study, we solved the problem of VLS early histologic diagnosis using second harmonic generation (SHG) microscopy [8]. SHG microscopy is a powerful tool for collagen fibers imaging with high sensitivity, specificity, and submicron resolution [9]. It allowed to estimate the organization of collagen fibers in normal and changed vulva dermis and to identify four degrees of VLS lesions: initial, mild, moderate, and severe [8]. This classification forms the basis of this study.

Optical coherence tomography (OCT) is a rapidly developing, non-invasive, label-free method of visualizing biological tissue structure, operating in real time with resolution up to several micrometers to 1.5-mm depth [10]. OCT is already used for many years to assess skin conditions in various diseases. In 2021, a group of researchers from King’s College London showed OCT effectiveness for assessing inflammatory skin diseases [11], but VLS was not studied. Our group successfully demonstrated earlier OCT application for non-invasive diagnostics of blood and lymphatic vessels state in VLS [12]. We use cross-polarization OCT (CP OCT) method in this study to identify non-invasively OCT signs of dermal lesion four degrees in VLS.

The CP OCT method is sensitive to changes of light polarization in tissues. Changes of polarization plane in biological tissues may be caused by double refraction or by microscopic scattering anisotropy [13, 14]. This allows to detect anisotropic tissue structures which longitudinally exceed transverse dimensions significantly [15]. Dermis collagen fibers are an example of these structures.

To assess dermis structure in VLS objectively, demonstration of absolute values characteristic of the tissue is necessary. One of these values obtained using the OCT signal is the attenuation coefficient, or decrease (attenuation) rate depending on how deep the OCT signal penetrates the tissue. Attenuation coefficient calculation allows to assess non-invasively changes of tissue morphology leading to changes in optical properties like absorption or probing radiation scattering. Attenuation coefficient calculation converts OCT signal initial values depending both on tissue properties and OCT device features into characteristics of the tissue itself, which will be repeated regardless of the device used [16, 17]. The method of calculating OCT attenuation coefficient is increasingly used to characterize tissues providing additional contrast to structural OCT images [17]. Thus, its effectiveness was demonstrated in studying skin burn scars [16, 17], age-related skin changes [18], as well as melanoma [19] and basal cell carcinoma [20].

In our group paper recently published, it was shown that collagen fibers undergo significant specific architectural changes at different VLS stages [8], and hence their scattering and polarization properties change. Consequently, it is reasonable to assume that different degrees of dermal lesions have specific values of signal attenuation in co- and cross-polarization. This will allow to identify non-invasively degrees of dermal lesions in VLS, including the initial degree.

**The aim of the study** is to identify different degrees of dermal lesions in vulvar lichen sclerosus using OCT cross-polarization method based on attenuation coefficient to detect disease early manifestations and to monitor the effectiveness of treatment.

## Materials and Methods

### Patients’ characteristics

The vulvar skin study was performed in the 1^st^ Gynecological Department of the Nizhny Novgorod Regional Clinical Hospital named after N.A. Semashko (Russia) from March 2018 to February 2020. During this period, 10 patients were selected without vulvar pathology and 39 patients with VLS diagnosed clinically. The average age was 59 years of patients without vulvar pathology (44 to 68 years) and 58 years of patients with VLS (52 to 69 years). The OCT examination was performed on the inner surface of the labia minora, in the main lesion area. After OCT examination, incisional biopsy was performed. Previously, the patients used no topical corticosteroids and calcineurin inhibitors that could have caused morphological changes in the vulvar skin. After histological examination, patients with VLS were divided into 4 groups as per dermal lesion degree: initial (8 patients); mild (7 patients); moderate (9 patients); severe (15 patients).

Before OCT examination and biopsy, voluntary informed consent was obtained from each patient. The study was approved by the Ethical Committee of Privolzhsky Medical Research University (Nizhny Novgorod, Russia) (record No.17 dated October 11, 2019) and was performed in accordance with the Declaration of Helsinki (2013).

### Cross-polarization OCT system

To examine the dermis connective tissue state, a CP OCT device was used based on the spectral principle of signal reception. The device was developed at the Institute of Applied Physics of the Russian Academy of Sciences (Nizhny Novgorod, Russia) [21, 22]. The radiation source is a 3-mW power, 1310-nm central wavelength superluminescent diode. The system resolution is 10 μm axially and 15 μm transversally (in air). The device scanning speed is 20,000 A-scans per second. From each scanning point, a 3.4×3.4×1.25-mm 3D data array was obtained in 26 s. [23]. During real-time scanning, two conjugate images are generated: in co- and cross-polarization. The device is equipped with a flexible fiber-optic pencil-type probe for contact examination.

### Analysis of the obtained OCT data

The analysis included three stages: 1) visual analysis of structural CP OCT images; 2) development of color-coded attenuation coefficient charts and their subsequent visual analysis; 3) quantitative and statistical evaluation of attenuation coefficients in co- and cross-polarization.

#### First stage

Two-dimensional CP OCT images (b-scans) were analyzed visually to identify signal parameters characteristic of dermis with different lesion degrees.

#### Second stage

An effective way to visualize attenuation coefficients is to represent charts in the *en face* plane, i.e. as attenuation coefficient parametric images [24]. The obtained color-coded charts were based on the distribution of coefficient values for OCT images in co- and cross-polarization. For this purpose, a 3D array of OCT data was converted into 2D *en face* images, which were plotted at two depths of 0 to 350 and 350 to 700 μm from the dermal-epidermal junction. Based on numerical value scatter of optical coefficients, a universal color scale was selected for charts in co- and cross-polarization. The minimum and maximum values taken as blue and red pseudo-colors, respectively, were set considering the optimal color contrast (with 0 to 12 mm^–1^ optimal value).

#### Third stage

To quantify and statistically estimate the attenuation coefficient in co-polarization, a depth resolution approach was used. This approach was proposed by Vermeer et al. [25] and assumes that the backscattering coefficient is proportional to the attenuation coefficient with a constant ratio between them over the OCT depth range. In this study, we adopted the method modified by our group and described by Gubarkova et al. [26] as it considers noise with non-zero mean which is present in the distribution of OCT images absolute values. This allows to avoid the systematic error of attenuation coefficient estimation characteristic of method proposed by Vermeer et al. [25].

To calculate the values of attenuation coefficient in cross-polarization, we used the method of logarithmic signal linear fitting according to Kut et al. [27], since the differential equations describing the signal propagation in co-polarization are not valid for the signal in cross-polarization, and therefore, the method based on their solution cannot be applied directly to the signal in cross-polarization.

Both attenuation coefficients were analyzed at depth ranges from dermal-epidermal junction 0 to 350 μm (subepidermal zone) and 350 to 700 μm (deep zone). The depth range selection depended on dermis varying depth and destruction degree.

### Histological examination

**Figure 1.**
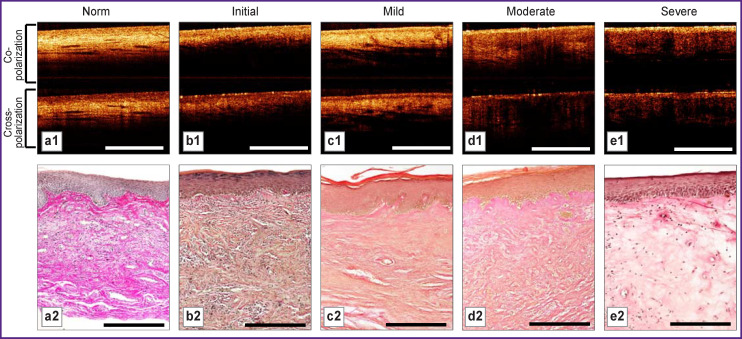
Structural OCT images (b-scan) and histological images of normal vulvar skin (norm) and skin in vulvar lichen sclerosus with different degrees of dermal changes (initial–severe): a1, b1, c1, d1, e1 — structural OCT images in co- and cross-polarization (1.5-mm bar); a2, b2, c2, d2, e2 — histological images, van Gieson’s picrofuchsin staining (300-μm bar)

Histological evaluation was aimed to verify CP OCT findings. Dermis overall histological structure was examined using Van Gieson’s picrofuchsin staining: collagen fibers turned crimson-red, cell nuclei turned black, and other tissue elements turned yellow (Figure 1 (a2–e2)). This staining is more informative than standard hematoxylin and eosin staining when studying collagen-rich connective tissue. Histological preparations were analyzed using transmitted light imaging automated system EVOS M7000 (Thermo Fisher Scientific Inc., USA). The histologic diagnosis was confirmed by two independent pathologists.

### Statistical analysis

Statistical data were processed using software IBM SPSS Statistics 26 (USA). To identify statistically significant differences between several study groups, nonparametric Kruskal–Wallis test was used with Bonferroni correction for multiple hypothesis testing. The results were considered statistically significant if p<0.05. The attenuation coefficient values were expressed as Me [Q1; Q3], where Me is the median, Q1 and Q3 are the 25^th^ and 75^th^ percentiles, respectively.

## Results and Discussion

### CP OCT structure of normal vulvar skin

At structural OCT-images, normal vulvar skin structure is layered: the first layer corresponds to the epidermis characterized by OCT signal of low intensity; the second layer is the dermis with higher signal level. The border between the epidermis and dermis is contrasting, with higher contrast in the cross-channel (Figure 1 (a1)). Elongated inclusions with low OCT signal are observed in the dermis, which are lymphatic vessels [28].

**Figure 2.**
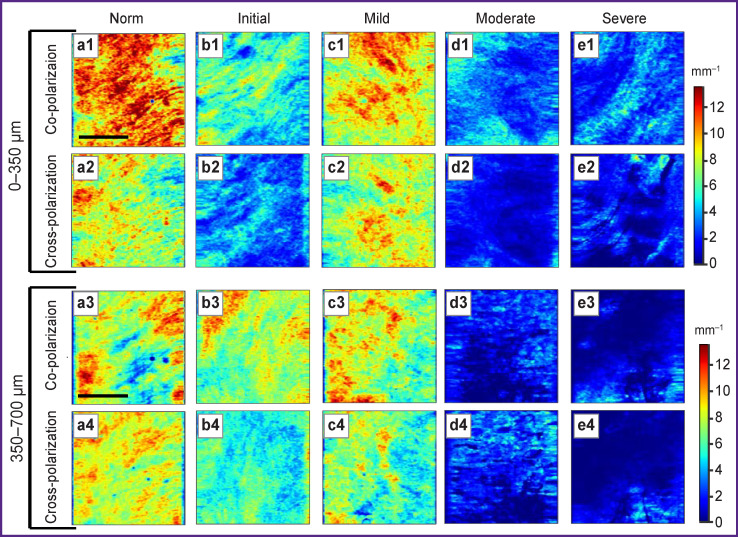
Images of color-coded attenuation coefficient OCT charts in co- and cross-polarization of normal vulvar skin (norm) and in vulvar lichen sclerosus with different degrees of dermis changes (initial–severe) at 0–350 and 350–700-μm depths from dermal-epidermal junction Color scales are located to the right of color-coded charts *en face*. Bar size at OCT charts is 1.5 mm

Color-coded attenuation coefficient charts in the subepidermal layer (0–350 μm) have different color palettes: red-yellow in the co-channel (Figure 2 (a1)), and yellow-green in the cross-channel (Figure 2 (a2)). In the deep dermis layer (350–700 μm), the attenuation coefficient charts both in co- and cross-polarization channels are represented by a yellow-orange palette (Figure 2 (a3, a4)).

Histological examination with Van-Gieson’s staining demonstrates an ordered structure of the dermis. Short collagen bundles are observed in the subepidermal layer, with thickness increasing from subepidermal to deep dermis (see Figure 1 (a2)).

### Dermis CP OCT structure at initial lesion degree

Even with initial degree of dermal lesions, changes of structural OCT images are significant. Dermal-epidermal junction contrast is reduced and dermis OCT signal intensity in co- and cross-polarization channels is lower than for normal state (Figure 1 (b1)). The dermis lacks low-signal slit-like structures (lymphatic vessels).

In the subepidermal zone, color-coded attenuation coefficient charts in co- and cross-polarization channels are represented by a green-blue palette with yellow inclusions (Figure 2 (b1, b2)), differing from normal state. In dermis deep zone, the co-channel shows a red-green attenuation coefficient palette indistinguishable from normal state (Figure 2 (b3)), and the cross-channel shows a green-blue palette different from normal state (Figure 2 (b4)).

Histological examination of dermis subepidermal layer shows thin, elongated collagen bundles with enlarged spaces between them due to edema and cellular inflammatory infiltrate (see Figure 1 (b2)). The dermis lesion depth was 200–250 μm, so changes at attenuation coefficients charts are observed only in the dermis upper layer.

Decrease of the dermis OCT signal intensity and hence of attenuation coefficient values at initial dermis lesion occurs due to decrease of collagen bundles arrangement density as a result of edema. Previously demonstrated results on low level of dermis OCT signal in psoriasis as a result of inflammatory reaction (vasodilation, cellular infiltration and interstitial edema) [29] are fully consistent with our data.

### Dermis CP OCT structure at mild lesion degree

It is important to note that in the group of VLS patients with mild degree of tissue damage, structural OCT images visualization is similar to that of normal vulva. Epidermis structure is not damaged. The dermal-epidermal border is more contrasting in the cross-channel than in the co-channel. Dermis signal is close to normal state (Figure 1 (c1)), but decrease is observed of slit-like structures number in the dermis.

Both in co- and cross-polarization channels, the color palette of attenuation coefficients is represented in yellow-green tones with red inclusions and is indistinguishable from the normal state. However, it differs sharply from both initial and moderate lesion stages (Figure 2 (c1, c2)). Deep zone coefficients retain a yellow-green palette with red inclusions (Figure 2 (c3, c4)).

Besides, the dermis histological picture differs significantly from normal state. It is represented by densely arranged short and thickened bundles of collagen fibers. No edema or inflammatory infiltrate are observed. The lesion area lies just under the epidermis and replaces the normal papillary dermis to a 320–350-μm depth (Figure 1 (c2)).

Increased attenuation coefficients compared to the initial lesion are due to dense arrangement of thickened collagen bundles, intensely scattering back the probing OCT radiation and generating a high OCT signal [30].

### Dermis CP OCT structure at moderate lesion degree

At moderate lesion degree, structural OCT images show a higher epidermis signal and a sharply lower dermis signal compared to dermis milder lesions (initial, mild). Besides, dermis signal drop in the cross-channel is stronger than in the co-channel. Epidermis signal is present, dermal-epidermal border is contrasting (Figure 1 (d1)).

Both in the co- and cross-polarization channels, attenuation coefficient charts are represented by a blue palette in the subepidermal zone (Figure 2 (d1, d2)), by a sky-blue and dark blue palette in the deep zone (Figure 2 (d3, d4)), this characteristically distinguishing this degree from normal state, initial and mild skin lesion in VLS.

Histological preparations stained with Van Gieson’s picrofuchsin show loss of dermis fibrous structure and formation of homogeneous masses with mild edema and inflammatory lymphocytic infiltrate (Figure 1 (d2)). The lesion zone affects both the subepidermal and deep dermis.

Although standard histological methods demonstrate dermis homogenization, high-resolution SHG microscopy shows that this area contains a large number of extremely thin collagen fibers [8]. This accounts for decrease of dermis scattering and anisotropic properties in near infrared range, and hence of attenuation coefficients both in OCT co- and cross-polarization channels.

### Dermis CP OCT structure at severe lesion degree

Structural OCT images show epidermis high signal resulting from hyperkeratosis and a sharply low homogeneous dermis signal. This signal distribution creates a high contrast of dermal-epidermal junction both in the co- and cross-polarization channels (Figure 1 (e1)).

At color-coded attenuation coefficient images, a blue palette without contrasting inclusions prevails throughout the dermis both in the co- and cross-polarization channels (Figure 2 (e1–e4)), this being exactly characteristic of severe dermal lesion degree in VLS.

Histologically, this is explained by formation of homogeneous opaline-glass-like tissue with intense edema and slight inclusion of lymphocytes, also characterized by sharp decrease of blood vessels density (Figure 1 (e2)). Dermis homogenization depth ranged from 480 to 1200 μm.

### Quantitative analysis of OCT attenuation coefficients

**Figure 3.**
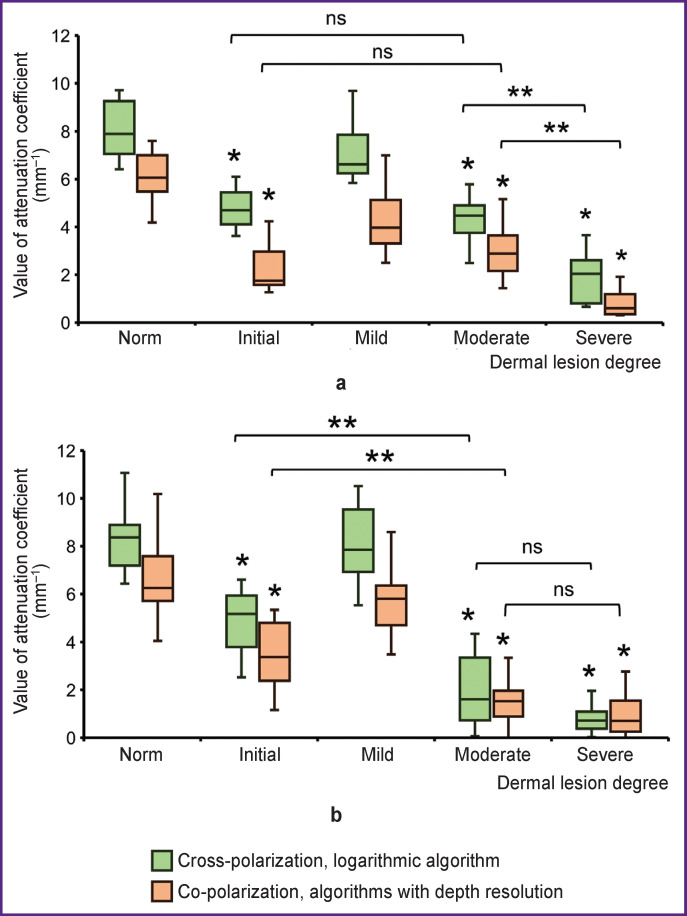
Quantitative parameters of attenuation coefficients in subepidermal (a) and deep (b) dermis layer (0–350 and 350– 700 μm, respectively) The data are represented as box-plots, where the central line represents the median; the upper and lower limits of the chart body are Q3 and Q1, respectively; the whiskers are values within 1.5 times the interquartile range Q1 to Q3; the extreme values are close to median 95% confidence interval. Nonparametric Kruskal– Wallis test with Bonferroni correction was used for statistical analysis; * groups differing from normal state with statistical significance (p<0.05); ** differing from each other (p<0.05); ns — no statistically significant differences between groups (p>0.05)

To objectively assess dermis state, attenuation coefficients in the co- and cross-polarization channels were quantitatively analyzed. Quantitative analysis results allowed to identify each dermal lesion degree from each other with statistical significance (Figure 3). All groups with lesions differed from normal vulvar skin with statistical significance, except for the group with mild degree of dermal lesion. A similar observation occurred as color-coded attenuation coefficient charts were analyzed visually. Perhaps if the observation group with mild dermal lesions is increased, we will get statistically significant results.

Below are shown quantitative parameters of attenuation coefficients allowing to identify dermal lesion degree from normal vulvar tissue with statistical significance (values are represented as Me [Q1; Q3]).

*1. Dermis initial lesion.*

Subepithelial zone (0–350 μm):

attenuation coefficient in the co-channel 4.69 [3.62; 5.45] mm^–1^, p<0.001;

attenuation coefficient in the cross-channel 1.75 [1.57; 2.97] mm^–1^, p<0.001.

Deep zone (350–700 μm):

attenuation coefficient in the co-channel 5.18 [3.79; 5.93] mm^–1^, p=0.001;

attenuation coefficient in the cross-channel 3.37 [2.38; 4.8] mm^–1^, p<0.001.

*2. Dermis mild lesion.*

No parameter difference with statistical significance.

*3. Dermis moderate lesion.*

Subepithelial zone (0–350 μm):

attenuation coefficient in the co-channel 4.47 [3.75; 4.89] mm^–1^, p<0.001;

attenuation coefficient in the cross-channel 2.88 [2.16; 3.64] mm^–1^, p<0.001.

Deep zone (350–700 μm):

attenuation coefficient in the co-channel 1.61 [0.73; 3.35] mm^–1^, p<0.001;

attenuation coefficient in the cross-channel 1.53 [0.89; 1.97] mm^–1^, p<0.001.

*4. Dermis severe lesion*.

Subepithelial zone (0–350 μm):

attenuation coefficient in the co-channel 2.04 [0.8; 2.6] mm^–1^, p<0.001;

attenuation coefficient in the cross-channel 0.59 [0.35; 1.19] mm^–1^, p<0.001.

Deep zone (350–700 μm):

attenuation coefficient in the co-channel 0.72 [0.38; 1.09] mm^–1^, p<0.001;

attenuation coefficient in the cross-channel 0.69 [0.26; 1.55] mm^–1^, p<0.001.

## Conclusion

For the first time, optical properties were characterized of initial, mild, moderate, and severe degrees of dermal lesion in VLS using CP OCT method. Quantitative parameters were identified for each lesion degree allowing to monitor changes dynamics of vulvar tissue during treatment. It was demonstrated that attenuation coefficient values of dermis OCT signal allow to distinguish dermal lesion initial degree in VLS from normal vulvar skin with statistical significance. This enables real-time, non-invasive diagnosis at the earliest stage of disease progression. Besides, we were unable to identify statistically significant differences between dermal lesion mild degree and normal skin based on OCT attenuation coefficients. This may be due to the small statistical sample.
